# Nitrogen Loss Estimation Worksheet (NLEW): An Agricultural Nitrogen Loading Reduction Tracking Tool

**DOI:** 10.1100/tsw.2001.282

**Published:** 2001-11-09

**Authors:** Deanna L. Osmond, Lin Xu, Noah N. Ranells, Steve C. Hodges, Roger Hansard, Stephen H. Pratt

**Affiliations:** Department of Soil Science, NC State University, Raleigh 27695-7619, USA

**Keywords:** river basin, regulation, nitrogen

## Abstract

The Neuse River Basin in North Carolina was regulated in 1998, requiring that all pollution sources (point and nonpoint) reduce nitrogen (N) loading into the Neuse Estuary by 30%. Point source N reductions have already been reduced by approximately 35%. The diffuse nature of nonpoint source pollution, and its spatial and temporal variability, makes it a more difficult problem to treat. Agriculture is believed to contribute over 50% of the total N load to the river. In order to reduce these N inputs, best management practices (BMPs) are necessary to control the delivery of N from agricultural activities to water resources and to prevent impacts to the physical and biological integrity of surface and ground water. To provide greater flexibility to the agricultural community beyond standard BMPs (nutrient management, riparian buffers, and water-control structures), an agricultural N accounting tool, called Nitrogen Loss Estimation Worksheet (NLEW), was developed to track N reductions due to BMP implementation. NLEW uses a modified N-balance equation that accounts for some N inputs as well as N reductions from nutrient management and other BMPs. It works at both the field- and county-level scales. The tool has been used by counties to determine different N reduction strategies to achieve the 30% targeted reduction.

## INTRODUCTION

Increasing levels of nitrogen (N), particularly nitrate-N, in surface waters throughout the world have led to the deterioration of coastal water quality[1]. This has occurred in the Gulf of Mexico, the Chesapeake Bay, Japanese and Danish coastal waters, and in other estuaries globally[2,3,4]. Despite the difficulty in assigning sources to nonpoint source pollution, the U.S. Environmental Protection Agency (USEPA) reports that the leading cause of water-quality impairment is agriculture[5].

N fertilizer is necessary to assure commercially viable yields. Overfertilization of N, however, not only increases N losses into surface water, but also reduces profits. The Albemarle-Pamlico Estuary, one of the most important estuaries in the U.S., has experienced hypoxic and anoxic conditions due to accelerated alga production from excess N, especially where the Neuse River empties into the estuary. This estuary, ringed by the Outer Banks, is very shallow and only completely flushes on average over a 3-year period. A rule to reduce N by 30% in the Neuse River passed in August of 1998. The Neuse Rule targets all sources of N, both point and nonpoint[6].

The Neuse Rule specifies that all agricultural producers in the Neuse River basin must either implement a standard best management practices (BMP) option or join a local area plan. Under the mandatory BMP option, producers must select one of the following standard alternatives: (1) a 15-m riparian buffer consisting of a 9-m tree buffer and 6 m of other vegetation, (2) nutrient management and a minimum 9-m vegetative buffer, (3) nutrient management and a minimum 6-m tree buffer, (4) nutrient management and controlled drainage, or (5) controlled drainage and either a 9-m grass or 6-m tree buffer. During a 1-year sign-up, 80% of all producers in the Neuse River basin registered to participate in the local area plan. As a condition to instituting the local area planning option, the use of a N-reduction tracking and accounting tool was mandated under the Neuse Rule. The Nitrogen Loss Evaluation Worksheet (NLEW) was developed to meet this requirement.

The NLEW tool was developed to serve a fivefold purpose: (1) estimate N loading from agricultural sources into the Neuse River during the baseline period of 1991 to 1995, (2) distribute goals for N reduction to local entities, (3) facilitate local BMP planning and implementation, (4) track implemented BMPs, and (5) account for reduction in N losses due to the implementation of BMPs throughout the basin. The development of the NLEW was possible due to an extensive research base that has been developed over the last 30 years in North Carolina.

Not all objectives could be met with one version of the NLEW. We had to design two versions: one that aggregates data by county and another that uses field-scale information. Objectives 1, 2, and 3 are met using the aggregate version of the NLEW, while objectives 4 and 5 are met by the field-scale version. We describe both versions of the NLEW below.

## N Loss Estimation Worksheet

Criteria for the development of the NLEW were established and include the following components:

1. Most N that is lost from a cropping system moves as soluble N.
2. Most of the available N in the soil system is either used by the crop or transported into the shallow groundwater.
3. Cropping systems are at semi-steady-state equilibrium with respect to background N dynamics.
4. The tool reflects biophysical processes that occur in the cropping system.
5. Inputs are readily available.

## Field-Scale Version

The NLEW tool was originally conceptualized to work at a field scale. Inputs needed for the field-scale accounting tool include dominant soil series, crop, field size (ha), current N fertilizer rate (kg/ha), realistic yield expectation (RYE) of the crop (kg/ha), cover crop species (optional), acreage of the cover crop, use of BMPs, and the acreage affected by the BMPs (Fig. 1). RYE values are defined as the average of the best 3 out of 5 years of yield and can be determined two ways: the producer can enter RYE values based on previous yield records or the RYE value can be obtained from the North Carolina RYE database[7].

Once the RYE value is obtained, the product of RYE times the N factor represents the N fertilization rate necessary to produce an optimum agronomic yield of a particular crop. In the NLEW, this N fertilization rate is referred to as the RYE N Rate. A range of N factors has been established for each of the major agronomic crops in North Carolina (Table 1)[8]. All the agricultural soils of North Carolina have been divided into soil management groups[9]. Soils are grouped based on physiographic region (coastal plain, piedmont, and mountains), drainage, productivity, texture, parent material, and landscape position. Each soil group has been assigned a N factor for every crop, based on soil-group characteristics. The N factors used in the NLEW are taken from the information contained in the N factor data table based on crop and soil management group.

If the RYE N Rate is less than the Current N Rate (the current applied N rate), the N fertilizer that the crop does not use is partitioned into Excess N (Excess N = Current N Rate – RYE N Rate). Of the N that is lost as Excess N, 95% is considered to be lost through subsurface processes, with the remaining 5% lost through surface transport. This partitioning of N was based on research conducted at North Carolina State University. It is assumed that the N lost via surface flow does not undergo any processes that would remove its delivery. N that infiltrates into the soil but is not utilized by the growing crop can be intercepted or transformed by specific BMPs.

Optimal crop production requires the application of an amount of N above that which can be retrieved by the crop in a given growing season. Agronomists have measured apparent fertilizer N use efficiency (NUE) values to determine the percentage of applied N recovered by the aboveground portions of the crop in a given year. NUE values were derived primarily from experiments conducted in North Carolina and also an extensive literature review (Table 2). The NUE values are based on the aboveground biomass. A semi-steady-state equilibrium with regard to N dynamics is assumed. The paucity of data on RYE and NUE values for less common crops such as strawberries and melons is a significant limitation to the current version of the NLEW; however, it can be easily updated as values become available.

The RYE N Rate is multiplied by the appropriate crop NUE value to determine the amount of Crop N Uptake. The remainder of the N is (1- Crop N Uptake). The remainder represents the N that is not absorbed by the crop and that can be leached into the shallow groundwater. This is the Subsurface N. Since the NLEW is not a complicated hydrologic model and does not attempt to account for all sources and losses of N (i.e., net mineralization and denitrification), the assumption is made that all the fertilizer N not used by the crop moves below the root zone. Lack of accounting for all sources and losses may produce some uncertainty in the results. However, ascribing ranges of loss due to denitrification or additions from mineralization also carries a high degree of uncertainty. Although the absolute value of N loss beneath the root zone appears to be higher in this simple worksheet method than measured values, the direction of the values and range are within experimental ranges.

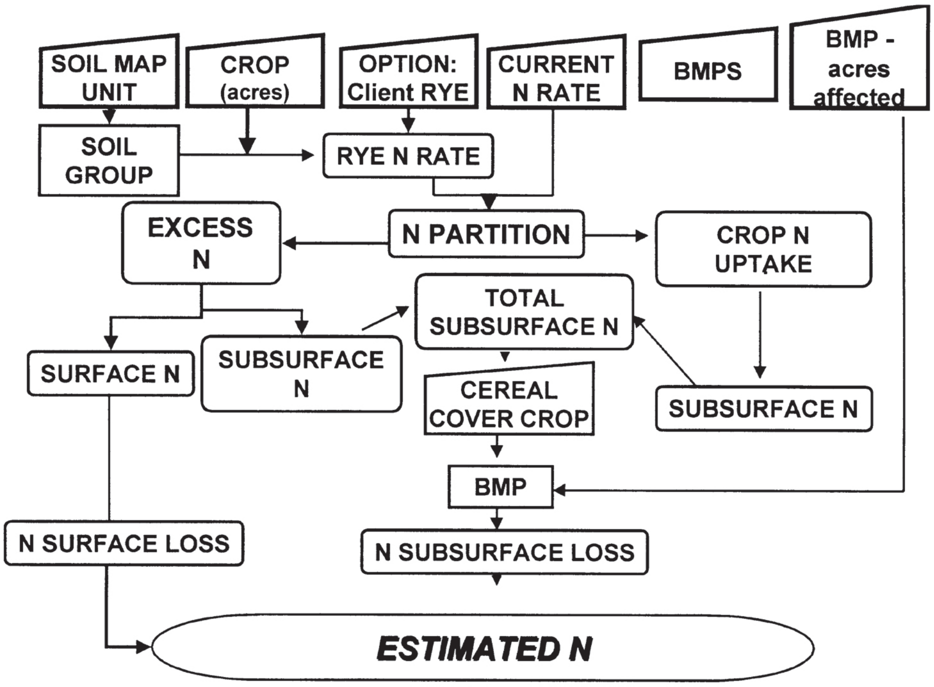

**FIGURE 1**. Schematic of field-scale NLEW.

**TABLE 1**
**N Factors for North Carolina Crops**

| Crop | Harvested portion | kg N/unit of yield | Unit of yield |
|---|---|---|---|
| Corn, grain | Grain | 0.018 – 0.022 | kg |
| Corn, silage | Silage | 5 - 6 | t |
| Wheat | Grain | 0.028 – 0.040 | kg |
| Rye | Grain | 0.028 – 0.040 | kg |
| Oat | Grain | 0.031 – 0.041 | kg |
| Barley | Grain | 0.029 – 0.033 | kg |
| Soybean | Grain | 0.058 – 0.067 | kg |
| Triticale | Grain | 0.023 – 0.027 | kg |
| Sorghum | Grain | 0.020 – 0.025 | kg |
| Bermudagrass | Hay | 20 - 25 | t |
| Tall fescue | Hay | 20 -25 | t |
| Small grains | Hay | 25 -30 | t |
| Cotton | Lint | 0.06 – 0.12 | kg |

The two subsurface N sources — consisting of excess applied fertilizer and fertilizer not utilized by the crop — are summed. If a cover crop is planted, the NLEW tool assumes that some of this excess N is absorbed by the unfertilized cereal cover crop. Much of the N absorbed by the cover crop will be released through mineralization to the subsequent crop. Thus, the N-reducing value assigned to the cover crop is the N that is removed from the system and not released during the subsequent growing season (Table 3). In order to receive N-reducing credit for cereal cover crops, the crop must be planted by November 30 and killed no earlier than March 31 in the coastal plain and April 10 in the piedmont.

Research at North Carolina State University has demonstrated that riparian buffers can reduce 85 to 95% of the subsur-

**TABLE 2**
**Fertilizer NUE for Selected Crops in North Carolina**

| Crop | N Use Efficiency (%) |
|---|---|
| Bermuda Grass | 75 |
| Flue-cured Tobacco | 50 |
| Burley Tobacco | 40 |
| Corn - Coastal Plain | 55 |
| Corn – organic soils | 40 |
| Corn – sandy soils | 40 |
| Corn – Piedmont, Conventional till | 40 |
| Corn – Piedmont, No-till | 55 |
| Sweet Potato | 40 |
| Cotton | 50 |
| Wheat | 45 |

**TABLE 3**
**BMP Reduction Efficiencies for N**

| Best Management Practice | N Reduction, % |
|---|---|
| Waster control structures (flashboard risers) | 40 |
| Riparian buffers | |
| 15 m width = 9 m trees and 6 m vegetation | 85 |
| Minimum 6 m trees | 75 |
| Minimum 9 m vegetation | 65 |
| Minimum 9 m vegetation | 40 |
| Cover crop | |
| Rye & Triticale | 15 |
| Oats & Barley | 10 |
| Wheat | 5 |

face N flowing into ditches and streams[10]. Controlled drainage structures can reduce this shallow groundwater pool by 40%. In the NLEW, the subsurface N can be affected by either of these BMPs. Because the acreage affected by these BMPs may not be the same as the field size, the area affected by the BMP must be determined. Subsurface N is multiplied by (1-% N Reduction by BMP), which gives the amount of N remaining in the subsurface N pool (N Subsurface Loss)(Table 3). The remaining N Subsurface Loss is added to the N Surface Loss to yield the Estimated N Loss. This designation of Estimated N does not necessarily represent N loading at the edge of field or stream loading N loss, but rather the end of the accounting process for the field.

## Aggregate Version

Under the Neuse Rules, historical losses of N from agricultural land uses had to be reconstructed for 1991 to 1995. There was insufficient historical field data to use the field-scale version of the NLEW. An aggregate version of the NLEW had to be developed to meet this need. The aggregate NLEW has coarser-scale inputs, but the basic structure of the tool is identical to the field-scale version (Fig. 2).

The number of acres in a specific soil management group is determined by the overlay of digital soil maps with 1993 land coverage data and basin boundaries. This overlay produces the

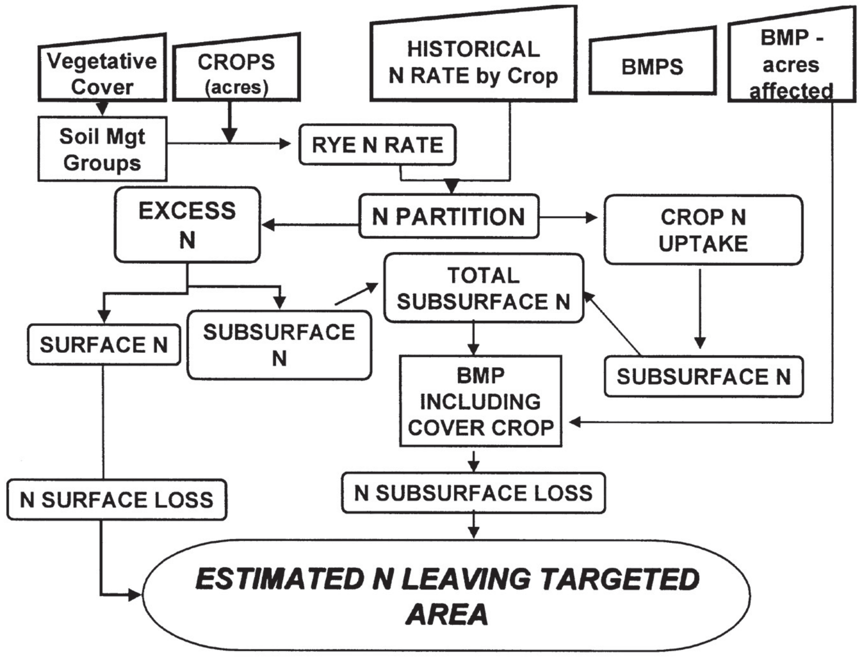

**FIGURE 2**. Schematic of aggregate NLEW.

soil series found within the local area (usually a county) and the number of acres of each soil series. The soil series and their acreages are then aggregated into their respective soil management group. Land coverage data may not be exact. Therefore, the acreage of the soil management groups can be adjusted within the computer program.

The crops and their acreage are then entered. These crop data were provided by the local agency personnel, most of whom obtained them from North Carolina Agricultural Statistics data for the 1991 to 1995 period. The crops are proportionally distributed across soil management groups based on acreage. Again, the crop distribution by acres can be adjusted within the computer program based on the experience of the personnel. For example, some crops may not be grown on a particular soil type. RYE values are then calculated for each soil management group and each crop.

Once the RYE is obtained, it is multiplied by a N factor. This multiplied value is the N fertilization rate (RYE N Rate) necessary to produce an optimum yield of that particular crop. The applied average fertilizer rate (Historical N Rate by Crop) for the period of 1991 to 1995 is entered for each crop. Historical N rates were obtained from agency personnel's best judgment. Historical fertilizer use data does not exist in North Carolina.

If the aggregate RYE N Rate is less than the aggregate Historical N Rate (the historical applied N rate), the extra N will be partitioned into Excess N; that is, the N fertilizer that the crop cannot use. The Crop N Uptake is aggregated by crop type. This means that the aggregate RYE N Rate for each crop is multiplied by 1- $NUE_{crop}$ and then summed. In other words, $\Sigma$(RYE N $Rate_{crop}$ (1- $NUE_{crop}$)) is the N that is not absorbed by the crop. This excess N can be leached through the soil and into the shallow groundwater. At this point the aggregate version of the NLEW proceeds exactly like the field-scale version in that the amount and types of BMPs can decrease subsurface N losses.

## RESULTS AND DISCUSSION

An easy-to-use computerized tool has been developed for both NLEW versions[11,12]. The NLEW was programmed in Delphi and can be installed on all IBM-compatible personal computers running Windows95 and all subsequent versions. The NLEW programs will not work on Linux or Apple computers.

Sequential runs of the aggregate NLEW tool have allowed agricultural agency staff to conduct pre- and post-BMP assessments of N losses. All inputs and outputs can be stored and also printed. Using the baseline period of 1991 to 1996, each county-level local area committee has determined the types and extent of the BMPs that they will implement to reduce N by 30%. As can be seen in Table 4, there are many different strategies being used by the counties to reduce their N losses. Some counties, such as Orange, are relying entirely on conversion to urban areas to reduce the impact of N losses from agriculture. Other counties, such as Lenoir, are using all the available BMPs. Without the aggregate version of the NLEW, all producers in the Neuse River basin would have been forced to use the mandatory BMPs. County-level staff has found the NLEW user-friendly and able to allow different strategies to be compared and tested.

The NLEW tool will continue to be used to track and account for N reductions by the agricultural community in the Neuse River basin as long as the river basin is regulated. We will con-

**TABLE 4**
**Strategies of Selected Counties for Reducing N by 30%**

| County | Water Control Structure (ha*) | Buffer: 6m (ha*) | Buffer: 9 m (ha*) | Buffer: 15 m (ha*) | Cropland Loss (ha) | Nutrient Management (ha) |
|---|---|---|---|---|---|---|
| Carteret | 6,970 | | | | | 9,269 |
| Johnston | | | 105 | 220 | 4,400 | 2,440 |
| Jones | | | | 32 | | 20,500 |
| Lenoir | 900 | 168 | 369 | 3,194 | | 410 |
| Orange | | | | | 1,070 | |
| Wayne | 74 | 305 | 329 | | | 46,511 |
| Wilson | | | | 491 | 2,418 | 2,870 |

* = drainage area tinue to assess the usefulness of the NLEW to track and account for N reductions during the next several years.

## CONCLUSIONS

Regulation of watersheds and river basins will continue to expand, particularly as Total Maximum Daily Loads are increasingly phased into use. Water quality monitoring to determine pollutant reductions, may not, however show progress, especially in the short term. Changes in water quality resulting from the implementation of BMPs can be determined by monitoring the particular resource of interest. Documentation of the magnitude of water-quality improvements from changes in land management is critical to ensure the efficacy of the selected BMPs. Historically, it has been difficult to demonstrate the relationship between land treatment and water quality changes, in part due to a lack of resources and well-designed water quality and land treatment monitoring efforts. However, monitoring the water resource alone is insufficient to document a cause-and-effect relationship between changes in water quality and changes in land treatment and land use[13]. To ascribe changes in water quality to land treatment, it is often necessary to intensively monitor and document both changes in water quality and changes in land use and land treatment over an extended period of time (at least 4 to 8 years). Land-based data requirements include detailed, timely, and site-specific information about land treatment practices and land use changes[14]. This type of water quality and land use monitoring is very expensive and often requires decades to show water-quality changes, particularly if the monitoring scale is large (e.g., a river basin).

In the Neuse River basin, regulators understood that water-quality monitoring to demonstrate reductions in N from agriculture would not be feasible within the 5-year time frame under which the rules operate. The NLEW is being used in lieu of water-quality monitoring to track the agricultural sector's implementation of BMPs and potential reduction of N losses into the Neuse River basin drainage area. Without such a tool, producers would have had few options in order to be in compliance with the Neuse Rule.

| | | |
|---|---|---|
| Received: | July | 18, 2001 |
| Revised: | September | 17, 2001 |
| Accepted: | October | 5, 2001 |
| Published: | November | 9, 2001 |

## BIOSKETCH

**Deanna Osmond** is an Associate Professor and Extension Specialist in the Soil Science Department of North Carolina State University, working in the area of water quality and production agriculture. Her extension and research activities promote appropriate technologies and siting of those technologies to reduce agricultural pollutants.
